# Artificial Intelligence-Enabled Electrocardiography Predicts Left Ventricular Dysfunction and Future Cardiovascular Outcomes: A Retrospective Analysis

**DOI:** 10.3390/jpm12030455

**Published:** 2022-03-13

**Authors:** Hung-Yi Chen, Chin-Sheng Lin, Wen-Hui Fang, Yu-Sheng Lou, Cheng-Chung Cheng, Chia-Cheng Lee, Chin Lin

**Affiliations:** 1 Department of Internal Medicine, Tri-Service General Hospital, National Defense Medical Center, Taipei 114, Taiwan; frank110tw@gmail.com; 2 Division of Cardiology, Department of Internal Medicine, Tri-Service General Hospital, National Defense Medical Center, Taipei 114, Taiwan; littlelincs@gmail.com (C.-S.L.); chengcc@mail.ndmctsgh.edu.tw (C.-C.C.); 3 Department of Family and Community Medicine, Tri-Service General Hospital, National Defense Medical Center, Taipei 114, Taiwan; rumaf.fang@gmail.com; 4 Graduate Institute of Life Sciences, National Defense Medical Center, Taipei 114, Taiwan; chaos53438@gmail.com; 5 School of Public Health, National Defense Medical Center, Taipei 114, Taiwan; 6 Planning and Management Office, Tri-Service General Hospital, National Defense Medical Center, Taipei 114, Taiwan; lcgnet@gmail.com; 7 Division of Colorectal Surgery, Department of Surgery, Tri-Service General Hospital, National Defense Medical Center, Taipei 114, Taiwan; 8 School of Medicine, National Defense Medical Center, Taipei 114, Taiwan

**Keywords:** artificial intelligence, electrocardiogram, deep learning, ejection fraction, heart failure, cardiovascular disease

## Abstract

**BACKGROUND:** The ejection fraction (EF) provides critical information about heart failure (HF) and its management. Electrocardiography (ECG) is a noninvasive screening tool for cardiac electrophysiological activities that has been used to detect patients with low EF based on a deep learning model (DLM) trained via large amounts of data. However, no studies have widely investigated its clinical impacts. **OBJECTIVE:** This study developed a DLM to estimate EF via ECG (ECG-EF). We further investigated the relationship between ECG-EF and echo-based EF (ECHO-EF) and explored their contributions to future cardiovascular adverse events. **METHODS:** There were 57,206 ECGs with corresponding echocardiograms used to train our DLM. We compared a series of training strategies and selected the best DLM. The architecture of the DLM was based on ECG12Net, developed previously. Next, 10,762 ECGs were used for validation, and another 20,629 ECGs were employed to conduct the accuracy test. The changes between ECG-EF and ECHO-EF were evaluated. The primary follow-up adverse events included future ECHO-EF changes and major adverse cardiovascular events (MACEs). **RESULTS:** The sex-/age-matching strategy-trained DLM achieved the best area under the curve (AUC) of 0.9472 with a sensitivity of 86.9% and specificity of 89.6% in the follow-up cohort, with a correlation of 0.603 and a mean absolute error of 7.436. In patients with accurate prediction (initial difference < 10%), the change traces of ECG-EF and ECHO-EF were more consistent (R-square = 0.351) than in all patients (R-square = 0.115). Patients with lower ECG-EF (≤35%) exhibited a greater risk of cardiovascular (CV) complications, delayed ECHO-EF recovery, and earlier ECHO-EF deterioration than patients with normal ECG-EF (>50%). Importantly, ECG-EF demonstrated an independent impact on MACEs and all CV adverse outcomes, with better prediction of CV outcomes than ECHO-EF. **CONCLUSIONS:** The ECG-EF could be used to initially screen asymptomatic left ventricular dysfunction (LVD) and it could also independently contribute to the predictions of future CV adverse events. Although further large-scale studies are warranted, DLM-based ECG-EF could serve as a promising diagnostic supportive and management-guided tool for CV disease prediction and the care of patients with LVD.

## 1. Introduction

Left ventricular dysfunction (LVD) is a critical disease [1], leading to high mortality [2] and costs of care [3]. Asymptomatic LVD is present in 3–6% of the general population [4]. According to the latest guidelines from the American College of Cardiology and the American Heart Association, echocardiography is not only a screening tool for LVD but also a therapeutic target in patients with heart failure (HF). Evidence-based therapies of lifestyle modification, such as diet control, strength training, and medical management should be initiated as soon as LVD is detected to decrease major adverse cardiovascular events (MACEs) [5,6,7]. For the early detection of asymptomatic LVD, B-type natriuretic peptide (BNP) and N-terminal pro-brain natriuretic peptide (NT-pro-BNP) are widely used tests with suboptimal accuracy [8,9], which can be confounded by age, sex, and disease history, and they provide limited performance with areas under the curve (AUCs) of 0.6–0.8 [6,10]. Currently, the echocardiographic ejection fraction (ECHO-EF) is the most important indicator for future complication predictions [6].

With the rapid progression of deep learning models (DLMs) in multiple categories, recent studies have revealed more accurate motor imagery classification of EEG using multilayer perceptron neural networks [11]. In obstetrics, fetal heart rate data analysis by a random forest algorithm provides accurate information on the health state of the fetus [12]. In addition, immunology research was carried out on disease detection, including autoimmune diseases, immunological deficiency syndromes, cancer, mental health, bacterial infection, and many more [13]. In cardiology, Shah et al. used a machine learning model to recognize cardiac arrest risk and survival probability [14]. During the COVID-19 pandemic, the real-time diagnosis of COVID-19 was important and was assisted with DLM-based chest X-ray images with an accuracy of 99% [15,16,17,18].

Electrocardiography (ECG) is an inexpensive, noninvasive and widely used tool for multiple chronic cardiac disease screenings and evaluations. With the rapid progression of deep learning models (DLMs) on ECG [19], these models have expanded to multiple applications and achieved human-level performance, effectively detecting cardiac diseases with large annotated ECG datasets, including arrhythmia detection [20], dyskalemia [21,22,23], myocardial infarction [24,25,26], aortic dissection [27], thyrotoxic periodic paralysis [28], and digoxin toxicity [29]. Interestingly, current studies have started to use DLM to interpret chronic changes in ECGs, such as anemia [30], diabetes [31], conduction abnormality [32], future atrial fibrillation [33], and mortality prediction [34].

An application of AI-ECG for the prediction of the LV ejection fraction (LVEF) has been developed. Previous studies by Kwon et al. [35] applied deep networks using extracted features to predict LVEF from ECGs, achieving AUCs of 0.843 in internal validation and 0.889 in external validation and outperforming other networks of logistic regression and random forest. Cho et al. [36] used 12-lead direct ECG data to develop a new AI algorithm for ECG-EF prediction with an AUC of 0.913 in internal validation and 0.961 in external validation, demonstrating the possibility of single-lead ECG for LVD detection and high correlations between model-derived features and clinically utilized features, including heart rate, QT, interval, QRS duration, and T axis. Attia Z. et al. [4] trained a DLM to identify patients with EF < 35% with a high AUC of 0.932 using ECG alone. Most importantly, the patients had a higher risk of future heart function decline when classified with abnormal EF compared to those classified with normal EF. It has been suggested that DLMs could find ECG abnormalities before overt dysfunction is detected by echocardiography. These results have emphasized the rapid development of DLMs for the diagnosis of LVD.

Recently, a new hypothesis was raised that cardiovascular outcome was significantly associated with subtle ECG changes identified by DLMs [34]. Patients with higher DLM-predicted ECG age compared to their real chronological age were associated with a higher incidence of hypertension, CAD, or low ECHO-EF [37]. Hypertension, hyperlipidemia, type 2 diabetes mellitus, smoking, and obesity are common causes of cardiovascular diseases, which contribute to ECG abnormalities [38,39,40]. Patients who smoke have ECG presentations of an increased heart rate, frequent ectopic beats, and ischemic ST-T wave changes [41]. Hypertension contributes to diastolic left ventricular dysfunction, presenting with prolonged ventricular activation time, P-wave terminal force in V1, or P-wave dispersion [42,43,44]. Moreover, increased P-wave duration and PR intervals were found among patients with obesity [45]. These results suggest a critical role for unstructured data in the prediction of cardiovascular disease outcomes and inspired us to investigate the effects of residual differences between ECG-EF and ECHO-EF on future adverse outcomes.

In this study, we trained DLMs to estimate EF by ECG. Furthermore, by evaluating the difference between ECG-EF and ECHO-EF, we explored the capacities of ECG-EF on the prediction of future changes in LVEF. Finally, the diagnostic capacities of ECG-EF regarding the outcomes of CVD, including MACEs, cardiovascular death, heart failure death, and sudden arrhythmia death were investigated.

## 2. Methods

### 2.1. Data Source and Population

This research was ethically approved by the institutional review board of Tri-Service General Hospital, Taipei, Taiwan (IRB NO. C202105049). The electronic medical records (EMRs) of our hospital include digital ECG signals, and records collected between 1 January 2012, and 31 December 2019 were available. We identified a first-exam 12-lead ECG acquired in the supine position and at least one TTE obtained within seven days of the index ECG. The ECGs were acquired at a sampling rate of 500 Hz with a 10-s period using a Philips 12-lead ECG machine (PH080A, Philips Medical Systems, 3000 Minuteman Road Andover, MA 01810 USA) and stored using the MUSE data management system. Inadequate ECG or echocardiographic information was excluded, such as noise interference, leads dislodged or dislocated, and data loss of heart rate, EF, or left ventricular diameters. The remaining ECGs were annotated by TTE information and collected in this study. For patients with multiple ECG and TTE datasets meeting the criteria, the earliest pair was used for follow-up analysis. The DLM was trained via raw ECG traces.

**Figure 1:**
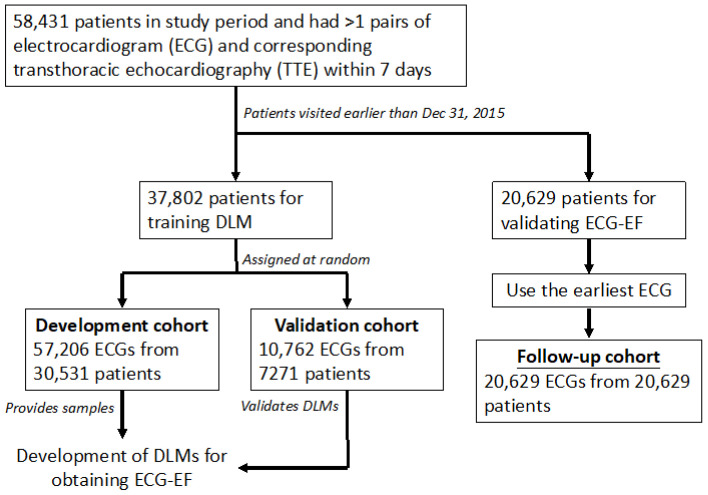
Development, validation, and follow-up cohort generation. Schematic strategy of the dataset creation and analysis. Cohort generation was based on different visiting dates. The follow-up cohort was divided into patients who visited earlier than 31 December 2015. The patients in the development and validation cohorts were assigned randomly and independently to the follow-up cohort, avoiding cross-contamination. Abbreviations: DLM: Deep learning model; ECG-EF: DLM predicted ejection fraction from ECG.

There were 58,431 patients with more than one pair of ECG-TTE datasets within seven days and corresponding demographic characteristics for the primary analysis. The ECG cohorts were divided into development, validation, and follow-up cohorts by date. Patients that visited earlier than 31 December 2015, were classified into the follow-up cohort, and the first pair of ECG-TTE data was used for DLM validation. The other 37,802 patients for training were assigned randomly to the development and validation cohorts. The development cohort included 57,206 ECGs from 30,531 patients used to provide samples, and the validation cohort included 10,762 ECGs from 7271 patients used to validate the DLMs. No patients were recruited into more than one group (Figure 1). Comprehensive 2D ECHO was available for all patients. Quantitative data were recorded at the time of the acquisition in a Philips image system^®^ (IntelliSpace Cardiovascular, version 3.1, Philips Medical Systems Nederland B.V., Veenpluis 4-6, 5684 PC Best, The Netherlands). EF was routinely acquired by experienced cardiologists or technicians using standardized methods. EF was determined using the Simpson method, M-mode, and the reported visually estimated EF. We traced the endocardial border in both the apical four-chamber and two-chamber views in end-systole and end-diastole. After dividing the left ventricular (LV) cavity into predetermined numbers of slices, LV volume and EF were calculated by an ECHO machine.

### 2.2. Observation Variables

The primary outcome was the ability of the DLM to identify patients with serial changes in EF and MACEs in the future. We chose an ejection fraction cutoff value of 35% or less owing to its clear-cut clinical and therapeutic importance. ECHO-EF was classified as low (≤35%), mildly reduced (35–49%), or normal (≥50%). MACEs included cardiovascular death and nonfatal events. We reviewed all causes of death and classified cardiovascular death into four categories. The first category was arrhythmia-related death, which was recorded as fetal ventricular arrhythmia, including VT or Vf. The second category was acute coronary syndrome-related death, including myocardial infarction (MI). The third category was stroke death. The fourth was heart failure-related death. Other nonfatal events were based on new diagnoses according to the corresponding International Classification of Disease, Ninth Revision and Tenth Revision (ICD-9 and ICD-10, respectively), including acute myocardial infarction (AMI, ICD-9 codes 410.x and ICD-10 codes I21.x), stroke (ICD-9 codes 430.x to 438.x and ICD-10 codes I60.x to I63.x), diabetes mellitus (DM, ICD-9 codes 250.x and ICD-10 codes E11.x), hypertension (HTN, ICD-9 codes 401.x to 404.x and ICD-10 codes I10.x to I16.x), and chronic kidney disease (CKD, ICD-9 codes 585.x and ICD-10 codes N18.x). Patients with at least two records of more than or equal to 126 mg/dL glucose or more than or equal to 6.5% HbA1c for six months were also considered to have DM. We defined at least 2 records of estimated glomerular filtration rates less than 60 mL/min/1.73 m^2^ as CKD. For each outcome, patients with corresponding diagnosis codes before the first ECGs in the follow-up group were excluded from follow-up analysis.

Echocardiography data were collected, including EF, chamber size, wall thickness, and pulmonary artery pressure. Additional patient characteristics and the nearest laboratory results within three days before and after enrollment were also obtained for risk evaluation and comparison. We used pre-existing codes and other ICD-9 and ICD-10 codes to define baseline comorbidities, including hyperlipidemia (ICD-9 codes 272.x and ICD-10 codes E78.x) and chronic obstructive pulmonary disease (COPD, ICD-9 codes 490.x to 496.x and ICD-10 codes J44.9).

### 2.3. The Implementation of the Deep Learning Model

The ECG-based ejection fraction (ECG-EF) was considered a function score of the heart, estimated by DLMs. The ECG12Net architecture with 82 convolutional layers and an attention mechanism was used to estimate EF. The technology details, such as model architecture, data augmentation, and model visualization, were described previously [22]. This neural network was based on dense connection technology to convey gradients from the last layers to the start layers. The major repeated module was a dense module with two convolutional layers, and the pooling layers were used to reduce the resolution of the feature map. Based on the same architecture, we trained a new DLM for ECG-EF. Each original ECG signal length was considered a 12 × 5000 matrix. We randomly cropped a length of 1024 sequences as input in the training process. For the inference stage, 9 overlapping lengths of 1024 sequences based on interval sampling were used to generate predictions that were averaged as the final prediction described previously [24,29].

We used an oversampling process to adequately recognize extreme EF values. The process was based on weights computed based on the prevalence of 20 equidistant intervals in the development cohort. However, we explored multiple oversampling strategies to maximize the model’s performance because ECG was related to sex and age. The first strategy was the standard oversampling process without any revision in each batch (no-match), and the initial parameters were generated at random. The second strategy was to use the first strategy with transfer learning via age estimation DLM (no match with transfer), which was based on a previous study [46]. The third strategy was to additionally match both sex and age (sex-/age-matched). This strategy was to ensure a balanced gender distribution in each batch and additionally consider the weight of age computed on the prevalence of 20 equidistant intervals in the development cohort. We trained the above three DLMs to compare their performance, similar to a previous study [31]. The network with the highest Pearson’s correlation (r) between estimated ECG-EF and ECHO-EF in the validation cohort was used. There were only three candidate DLMs in this study with the same optimization parameters as described in the next paragraph.

In our study, approximately 64% of the datasets were used for training the network. ECGs were fed to the DLM, and the network weights were updated using Adam optimization with standard parameters. The MXNet software package, version 1.3.0, was implemented in our deep learning model. We trained DLMs with a 36 minibatch size and used an initial learning rate of 0.001 (β_1_ = 0.9 and β_2_ = 0.999). The learning rate decayed by a factor of 10 each time the loss on the validation cohort plateaued after an epoch. The only regularization method for avoiding overfitting in this study was a weight decay of 10^−4^. To prevent the networks from overfitting, early stopping was performed by saving the network after every epoch and choosing the saved DLMs with the least loss in the validation cohort.

### 2.4. Statistical Analysis and Model Performance Assessment

Cohort characteristics are presented as numbers of patients, percentages, means and standard deviations. Two variations were compared using analysis of variance (ANOVA), Student’s *t*-test, or the chi-square test, as appropriate. The primary endpoint of this study was to develop a DLM network to predict ECHO-EF changes and MACEs between patients with an EF of 35% or less and those with an EF greater than 35%. The optimal DLM was selected based on the highest Pearson’s correlation (r) between ECG-EF and ECHO-EF. The performance of the DLMs was evaluated by mean absolute errors, calculated in both the validation cohort and follow-up cohort. The area under the curve (AUC), sensitivity (recall), specificity, precision, and F-measure are also presented. We used the confusion scatter plot and Pearson’s correlation coefficient to compare the correlations between the predicted ECG-EF and actual ECHO-EF.

Univariable and multivariable Cox proportional hazard models were used to evaluate the predictive ability of ECG-EF, actual ECHO-EF, and other characteristics regarding CVD-related outcomes, with standardized hazard ratios (HRs) and 95% conference intervals (95% CIs) for comparison. Time-dependent receiver operating characteristic (ROC) curves and Kaplan–Meier curves were plotted to compare outcomes between different ECG-EF cohorts. All statistical analyses were completed in R software, version 3.4.4. The significance level was set as *p* < 0.05.

## 3. Results

Supplementary Table S1 shows patient characteristics for the development, validation, and follow-up cohorts, which were different among these three cohorts except for BMI. Patients in the reduced ECHO-EF group were predominantly elderly and male (69.7% men aged 67.8 ± 15.9 years old vs. 49.0% men aged 65.8 ± 16.8 years old), combined with a more chronic history and comorbidities such as AMI, CAD, HF, AF, and CKD. We subsequently compared three different strategies in the validation cohort (Supplementary Figure S1). Performance comparison showed that ECG-EF predicted by DLM with sex/age matching had the highest crude correlation (r = 0.621) with ECHO-EF. We performed age-stratified analysis in gender-matched subgroups, and the weighted mean of the correlation was the highest between ECG-EF and ECHO-EF. Therefore, the ECG-EF was defined as the estimation of DLM with a sex-/age-matched strategy.

**Figure 2:**
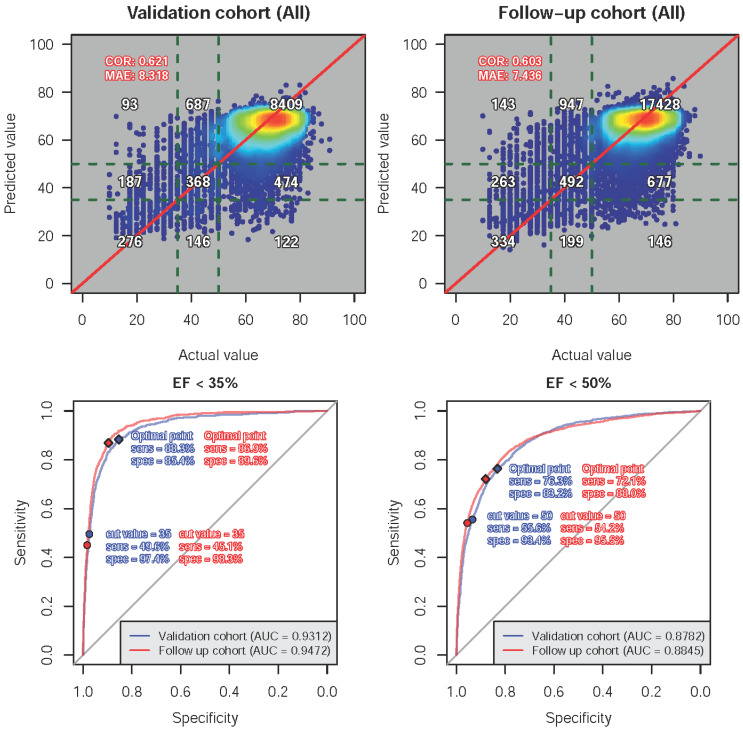
A comparison between actual EF and ECG-EF in the validation and follow-up cohorts. The confusion scatter plots (**top panel**) show the correlation (COR) and mean absolute error (MAE) between the actual value and predicted value based on the DLM trained using a sex-/age-matching strategy. ROC curves (**bottom panel**) demonstrate two cutoff points to calculate the sensitivities and specificities. The optimal point was based on the maximum Youden index in the validation cohort.

**Figure 3:**
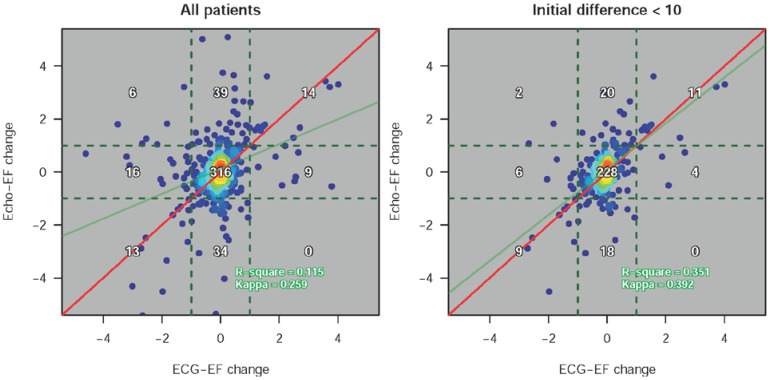
The relationship between ECHO-EF change and ECG-EF change in the follow-up cohorts. The changes were defined as the monthly changes based on linear regression with more than 3 points. Examples are shown in Supplementary Figure S2. The accurate cases with a <10% difference between the first ECHO-EF and ECG-EF were more consistent.

Figure 2 shows a comparison of actual ECHO-EF and ECG-EF in the validation and follow-up cohorts. Scatter plots revealed that the mean absolute errors of ECHO-EF versus ECG-EF were 8.318 and 7.436, respectively, with correlations of 0.621 in the validation cohort and 0.603 in the follow-up cohort. With two different clinical cutoff points of 35% and 50%, we generated ROC curves with sensitivities and specificities. The AUCs of ECG-EF to detect real ECHO-EF less than 35% or 50% were 0.9472 and 0.8845 in the follow-up cohort, respectively. The DLM had good prediction performance with a sensitivity of 86.9%/72.1% and a specificity of 89.6%/88.0% using the optimal cutoff points. Patients with more than 3 EEG-TTE pairs were further analyzed for the trend in EF change. As shown in Supplemental Figure S2, ECG-EF could predict the trend in monthly changes in actual ECHO-EF. Figure 3 demonstrates the relationship between ECHO-EF and ECG-EF. The variance (R-square) explained by ECG-EF for actual EF in the follow-up cohort was 0.115. Interestingly, if the initial difference between ECG-EF and ECHO-EF was less than 10%, ECG-EF had a higher correspondence with ECHO-EF, and the R-square increased to 0.351.

**Figure 4:**
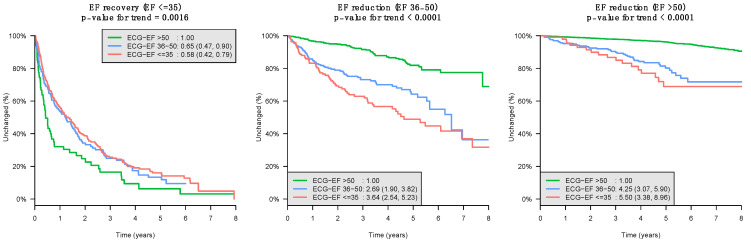
The comparison of ECHO-EF change (recovery or reduction) over time between different ECG-EF classifications. Long-term outcome of patients with a different echocardiographic EF at the time of initial classification (low, mildly reduced, or normal), stratified by the initial network classification. The ordinate shows the cumulative incidence of EF change, and the abscissa indicates years from the time of index ECG–TTE evaluation. Patients with normal ECG-EF served as the reference group. The left panel shows a faster ECHO-EF recovery when DLM defined the ECG-EF as normal (age- and sex-adjusted HR, 0.58 (95% CI, 0.42–0.79), *p* = 0.0016) compared with those with low ECG-EF. In contrast, the middle and right panels show the risk of future LV dysfunction when DLM defined the ECG-EF as low compared with those with normal ECG-EF (mild reduced: age- and sex-adjusted HR 3.64, 95% CI 2.54–5.23, normal: age- and sex-adjusted HR 5.50, 95% CI 3.38–8.96, all *p* < 0.0001). All analyses were performed based on a Cox proportional hazard model. EF: ejection fraction.

The estimation error was analyzed in terms of patient characteristics, as shown in Supplementary Figure S3. The patients with lower ECG-EF had larger heart sizes, higher pulmonary artery pressure, and a higher prevalence of HF/CKD/DM. All of these features are risk factors for MACEs. Figure 4 presents the primary outcomes of overall survival and comparisons between each ECG-EF group. If DLM predicted a patient with an ECG-EF greater than 50%, the recovery was significantly faster, and EF reduction was significantly slower than that in patients with an ECG-EF less than 35%. The secondary outcomes with complete stratified analysis are shown in Supplementary Figure S4. The same trend was observed in which patients with lower ECG-EF had poor prognoses and a high incidence of new-onset HTN, stroke, MI, DM, or CKD. These findings implied that ECG-EF could provide additional information about ejection fraction changes.

**Figure 5:**
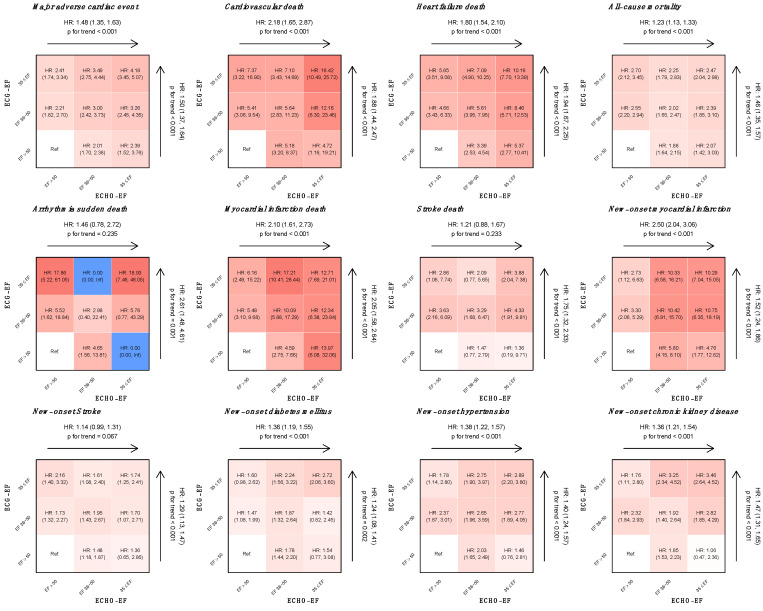
Risk matrices of different DLM predicted ECG-EF and the actual ECHO-EF on adverse outcomes. The hazard ratios (HRs) are based on the Cox proportional hazard model. Patients with ECG-EF ≤ 35% were significantly more susceptible to CV outcomes and new-onset comorbidities than patients with ECG-EF > 50%. The arrows represent the trend of risk as ECG-EF or ECHO-EF decreased. The color gradient represents the risk of the corresponding group, and nonsignificant results are shown in white.

We compared patients with abnormal ECG-EF to the healthy reference group and analyzed the hazard ratio (HR) of each outcome, as shown in Figure 5. The patients with false-positive detection by DLM (ECG-EF ≤ 35%) were significantly more susceptible to MACEs (HR: 1.50, 95% CI: 1.37–1.64), cardiovascular death (HR 1.88, 95% CI 1.44–2.47), heart failure death (HR 1.94, 95% CI 1.67–2.25), all-cause mortality (HR 1.46, 95% CI 1.35–1.57), arrhythmia sudden death (HR 2.61, 95% CI 1.48–4.61), myocardial infarction death (HR 2.05, 95% CI 1.58–2.64), and stroke death (HR: 1.75, 95% CI 1.32–2.33) compared to the true negative (ECG-EF > 50%) detection in patients with an actual ECHO-EF greater than 50%. Compared to ECHO-EF, the beneficial role of ECG-EF was demonstrated in the prediction of MACEs, HF death, all-cause mortality, sudden arrhythmia death, and stroke death. The ECG-EF also provided additional information on the prediction of new-onset comorbidities. The risk effect analysis of selected patient characteristics on primary outcomes by the Cox proportional hazard model is shown in Supplement Figure S5. In both univariate analysis and multivariate analysis, the previous comorbidities of cardiovascular disease, higher BNP level, and lower ejection fraction were significantly correlated with future EF reduction and MACEs. ECG-EF had a similar correlation or higher contribution than ECHO-EF to both the primary and secondary outcomes. The complete risk effect analysis of secondary outcomes is presented in Supplementary Figure S6, showing that ECG-EF is still significant for arrhythmia death, MI death, stroke death, new onset MI, stroke, DM, HTN, and CKD. Supplementary Figure S7 showed the performance for detection of decreased EF using BNP in validation and follow-up cohorts.

Table 1 shows the C-index comparisons of different models on CV-related outcomes. The C-indices of ECG-EF were significantly higher than those of ECHO-EF, which were 0.803, 0.662, 0.773, 0.825, and 0.648 for EF reduction, MACEs, CV death, HF death, and all-cause mortality, respectively. Even for secondary outcomes, the models had similar results. After we combined other factors, such as ECHO data or patient characteristics, with Models 1 and 2, the concordance indices of the new model were significantly better than those of the older model.

## 4. Discussion

In this study, we developed a DLM to accurately predict ECHO-EF by ECG with comparable results to previous studies [4,35,47], especially among patients with an initial difference of less than 10% between ECG-EF and ECHO-EF. Furthermore, the serial ECG-EF changes were correlated with the actual ECHO-EF changes, both in reduction and recovery. Most importantly, ECG-EF could independently predict MACEs, cardiovascular death, heart failure death, sudden arrhythmia death, stroke death, new-onset stroke, hypertension, and CKD based on the differences in ECG-EF and ECHO-EF. This result suggested that our ECG-EF DLM could be a promising integrated tool to evaluate cardiac status in addition to ECHO-EF. Moreover, previous studies clearly showed that EF reduction (<35%) developed within three years when discrepancies between ECHO-EF and ECG-EF existed over the follow-up periods of 8–10 years. Notably, apart from focusing on the recovery of LVEF in asymptomatic patients with LVD in their study, our study provides evidence that our AI ECG-EF anticipated significant changes in the recovery or reduction of ECHO-EF in patients with reduced or preserved EF, respectively.

Regular evaluation of cardiac function is important for patients with HF. Echocardiography is the gold standard for the evaluation of LVEF and the adjustment of medications in patients with HF. Moreover, it is a valuable tool for detecting several cardiovascular conditions, including asymptomatic structural heart problems, which have important prognostic implications [1,6,48]. The current echocardiography price is approximately 73 US dollars per examination, including physician, sonographer, and transducer costs [49]. If a patient received complete transthoracic echocardiography during hospitalization, the charge was 980 US dollars per patient per week [50]. Compared to echocardiography, AI ECG-EF is a convenient, inexpensive, and easily available tool that can be performed even in remote areas. Moreover, for those with an initial difference of less than 10% between ECHO-EF and ECG-EF, ECG-EF exhibited accurate prediction of LVEF, which is useful for long-term follow-up of patients with impaired LVEF.

Regarding the prediction of LVEF by 12-lead ECG raw data, our study evaluated 58,431 ECGs and achieved AUC performances of 0.93 and 0.9472 in the validation and follow-up cohorts, respectively, consistent with previous studies [4,35,47]. Table 2 summarizes the DLM performance in each study to detect LVD using ECG. Attia et al. [4] achieved a high AUC of 0.932 in detecting asymptomatic LVD by evaluating 97,829 ECGs. Moreover, they validated its performance in external populations [47,51]. Other studies have also developed a DLM to detect LVD with similar performance [35,36,52]. The most important contribution of this study was not only to present a similar performance compared to these papers but also to focus on revealing the prognostic value of the AI-enabled ECG model. In addition to the prediction of LVEF, the application of ECG-EF further significantly enhanced the predictive power of cardiovascular disease outcomes when integrating the information from ECHO-EF, full echocardiographic data, and patient characteristic data. Learning the subtle ECG characteristics associated with risk factors for cardiovascular disease, including DM, smoking, hyperlipidemia, and hypertension partly elucidates the ability of ECG-EF to predict cardiovascular disease outcomes. Changes in ion channel or transporter function secondary to the underlying cardiovascular disorder might alter the initiation or propagation of the cardiac action potential, leading to early presentation of abnormal ECG patterns, such as lower QRS maximum wave voltage, heart axis deviation, conduction delay, atrioventricular block, or P or T wave prolongation, all occurring before the manifestation of gross structural abnormalities [53,54,55]. In combination with the characteristic data from ECHO-EF and individual patients, our DLM extracts unstructured information, which is critical and exhibits synergistic effects on the prediction of cardiovascular disease outcomes.

Among patients with low ECHO-EF (<35%), the patients with low ECG-EF (<35%) exhibited the highest HR of MACEs, cardiovascular death and heart failure death. Currently, specific ECG patterns with evolving MI, defined as an ST elevation with T wave inversion and/or pathological Q waves in leads with ST elevation, are proposed to be associated with an increased rate of death from cardiovascular causes, recurrent MI, cardiogenic shock, or New York Heart Association (NYHA) class IV heart failure within one year [56]. Obscure myocyte lengthening, accounting for chamber dilation and remodeling [57,58], might be the underlying mechanism that changes the ECG early and induces a vicious cycle of myocardial injury and heart systolic function. Microscopic chamber and myocyte changes could contribute to subtle ECG presentations detected by DLM, which is beyond the human scale. Our data demonstrated the beneficial effects of combining DLM-based ECG-EF and ECHO-EF on the screening of high-risk patients with MACEs, cardiovascular death and heart failure death.

A low ECG-EF exhibited a high HR of sudden arrhythmia death in patients with an ECHO-EF of less than 35%. The causes of mortality in patients with reduced LV systolic function (EF < 40%) are largely due to ventricular arrhythmia [53,59]. Current AHA guidelines have proposed class I recommendations for implantable cardioverter-defibrillators (ICDs) in these patients to prevent sudden cardiac death [53]. Although further large-scale confirmatory studies are warranted, our study with ECG-EF identified patients with low ECHO-EF at high risk of sudden arrhythmia death, providing a novel and promising screening tool for ICD implantation. Interestingly, a low ECG-HF in patients with ECHO-EF greater than 50% was associated with a high HR of sudden arrhythmia death. Since the most common causes of sudden death are associated with MI [53,54,59], the undisclosed ischemic manifestations on ECG in patients with pending MI partly elucidate the high risk of sudden arrhythmia death in patients with normal ECHO-EF but low ECG-EF. Although low ECHO-EF in patients with MI has a high HR for death prediction, low ECG-EF provides the highest HR of MI death among patients with an ECHO-EF of 35–50%. Such evidence underscores the critical role of ECG-EF in death prediction in MI patients with mid-range LV systolic function.

Interestingly, we found that ECG-EF and ECHO-EF could be applied for the prediction of DM, hypertension, and chronic kidney disease (CKD), especially for the prediction of MI. As mentioned above, these cardiovascular diseases might exhibit subtle ECG presentations that are not easily identified by clinical physicians. Early detection of these diseases helps to provide preventive strategies, including lifestyle modification and conventional risk factor reduction, which could further reduce the disease and the concomitant economic burdens. Such evidence indicates that our ECG-EF not only predicts heart functional status but could also be exploited for the prevention of cardiovascular diseases.

There are some limitations of this study. First, this study was a retrospective study from one institution. Although ECGs were collected in both outpatient and inpatient settings, further community-based prospective studies are necessary to validate the accuracy and application of ECG-EF. Second, the ECG characteristics found by CNN cannot be ascertained. It applies a set of methods that allows the model to be created using raw data for automatic identification of the features and relationships. Further interpretation of the algorithm and explanation of deep learning are needed. Third, novel optimization techniques proposed recently were not applied in this study, such as the Whale Optimizer or chimp optimization algorithm. These optimizers could provide better performance and increase the reliability of the network while maintaining its capability [15,16,60]. Finally, the ECG-ECHO pairs were not simultaneously acquired. We collected all echocardiographic data seven days before or after the ECG exam, and 80% of the ECGs were collected within three days, restraining the errors related to temporal differences.

In conclusion, we developed a DLM from a large number of ECGs and echocardiographic data to accurately detect LVD and predict EF changes. Our DLM could be applied to identify asymptomatic patients with LVD for several applications, such as wearable devices and remote health care systems, which could help physicians initiate appropriate management for high-risk patients. Moreover, DLM-based ECG-EF analysis enhances the prediction of CV disease outcomes. Further large-scale and prospective studies are warranted to validate the clinical impact of diagnosis time reduction, the effect of early management, morbidity and mortality reduction, and cost-effectiveness. The DLM-based ECG-EF could serve as a promising diagnostic supportive and management-guided tool for CV disease prediction and the care of patients with LVD.

## Figures and Tables

**Table 1:** C-index comparisons of different models on CV-related outcomes.

Used Variables	EF Related Variables †			Full Echocardiography Data †		Full Characteristic Data †	
	ECHO-EF	ECG-EF	ECHO-EF + ECG-EF	Model 1 ※	Model 1 + ECG-EF	Model 2 ※	Model 2 + ECG-EF
**Primary outcomes**							
EF recovery	0.497	0.566 **	0.569 **	0.569	0.592 *	0.613	0.624
EF reduction	0.750	0.803 ***	0.811 ***	0.812	0.832 ***	0.817	0.834 ***
MACE	0.611	0.661 ***	0.664 ***	0.705	0.714 ***	0.723	0.730 ***
CV death	0.705	0.773 ***	0.777 ***	0.830	0.840 **	0.845	0.852 **
HF death	0.745	0.825 ***	0.821 ***	0.869	0.878 *	0.892	0.897
All-cause mortality	0.591	0.648 ***	0.650 ***	0.712	0.718 ***	0.748	0.752 ***
**Secondary outcomes**							
Arrhythmia death	0.654	0.824 **	0.822 **	0.897	0.906	0.904	0.912
MI death	0.792	0.793	0.822 **	0.861	0.862	0.876	0.876
Stroke death	0.596	0.702 ***	0.701 ***	0.792	0.809 *	0.813	0.826 *
New-onset MI	0.720	0.770 ***	0.778 ***	0.821	0.829 **	0.833	0.841 **
New-onset Stroke	0.565	0.613 ***	0.615 ***	0.657	0.664 ***	0.686	0.691 ***
New-onset DM	0.550	0.606 ***	0.605 ***	0.648	0.653 **	0.652	0.657 ***
New-onset HTN	0.567	0.631 ***	0.633 ***	0.694	0.699 ***	0.705	0.709 ***
New-onset CKD	0.585	0.630 ***	0.635 ***	0.678	0.685 ***	0.714	0.717 **

† The hypothesis test was based on the difference between each C-index and the first C-index in three parts (*: *p* < 0.05; **: *p* < 0.01; ***: *p* < 0.001). ※ variables included in Model 1: EF, LV-D, LV-S, IVS, LVPW, LA, AO, RV, PASP, and PE; variables included in Model 2: all variables included in Model 1, plus gender, age, BMI, AMI, stroke, CAD, HF, AF, DM, HTN, CKD, HLP, and COPD.

**Table 2:** Model performance comparison in current works.

	LVD Definition	AUCs	Sensitivity	Specificity	Future Outcomes
Attia, et al., (2019) [4]	EF ≤ 35%	0.932	86.3%	85.7%	EF reduction ≤ 35%
Kwon, et al., (2019) [35]	EF ≤ 40%	0.843 (Internal)	90.0%	60.4%	N/A
		0.889 (External)			
Attia, et al., (2019) [47]	EF ≤ 35%	0.911 (<1 year)	81.5%	86.3%	N/A
	EF ≤ 35%	0.918 (<1 month)	82.5%	86.8%	
Cho, et al., (2020) [36]	EF ≤ 40%	0.913 (Internal)	90.5%	75.6%	N/A
		0.961 (External)	91.5%	91.1%	
Attia, et al., (2021) [51]	EF ≤ 35%	0.820	26.9%	97.4%	N/A
Vaid, et al., (2021) [52]	EF ≤ 40%	0.94 (Internal)	89%	83%	EF reduction ≤ 35% Survival rate
		0.94 (External)	87%	85%	
	EF ≤ 35%	0.95 (Internal)	94%	83%	
		0.95 (External)	88%	87%	
This study	EF ≤ 50%	0.885	72.1%	88.0%	EF reduction ≤ 35% MACEs CV death CV complications
	EF ≤ 35%	0.947	86.9%	89.6%	

LVD: left ventricular dysfunction; AUCs: area under the curve; EF: ejection fraction; Internal: internal validation; External: external validation; MACEs: major adverse cardiovascular events.

## Data Availability

The data presented in this study are available on request from the corresponding author.

## Supplementary Materials

The following supporting information can be downloaded at: www.mdpi.com/article/10.3390/jpm12030455/s1, Figure S1: Performance comparison of DLMs trained by 3 different training strategies in validation cohort; Figure S2: The selected cases with more than 3 ECHO-EFs and ECG-EFs; Figure S3: The patient characteristics in different ECG-EF groups and real EF groups; Figure S4: The comparison between each ECG-EF group on secondary outcomes. Figure S5: Risk effect analysis of patient characteristics on primary outcomes; Figure S6: Risk effect analysis of patient characteristics on secondary outcomes; Figure S7: The performance for detection of decreased EF using BNP in validation and follow-up cohorts; Table S1: Corresponding patient characteristics and laboratory results of AD and non-AD records in the ECG dataset.
