# Supplementary material for: Artificial Intelligence-Enabled Electrocardiography Predicts Left Ventricular Dysfunction and Future Cardiovascular Outcomes: A Retrospective Analysis
Source: J Pers Med. 2022 Mar 13;12(3):455. doi: 10.3390/jpm12030455 (PMC8950054; doi:10.3390/jpm12030455)
Supplement: Supplementary file 1 [file jpm-12-00455-s001.zip › jpm-1580405-supplementary.pdf]

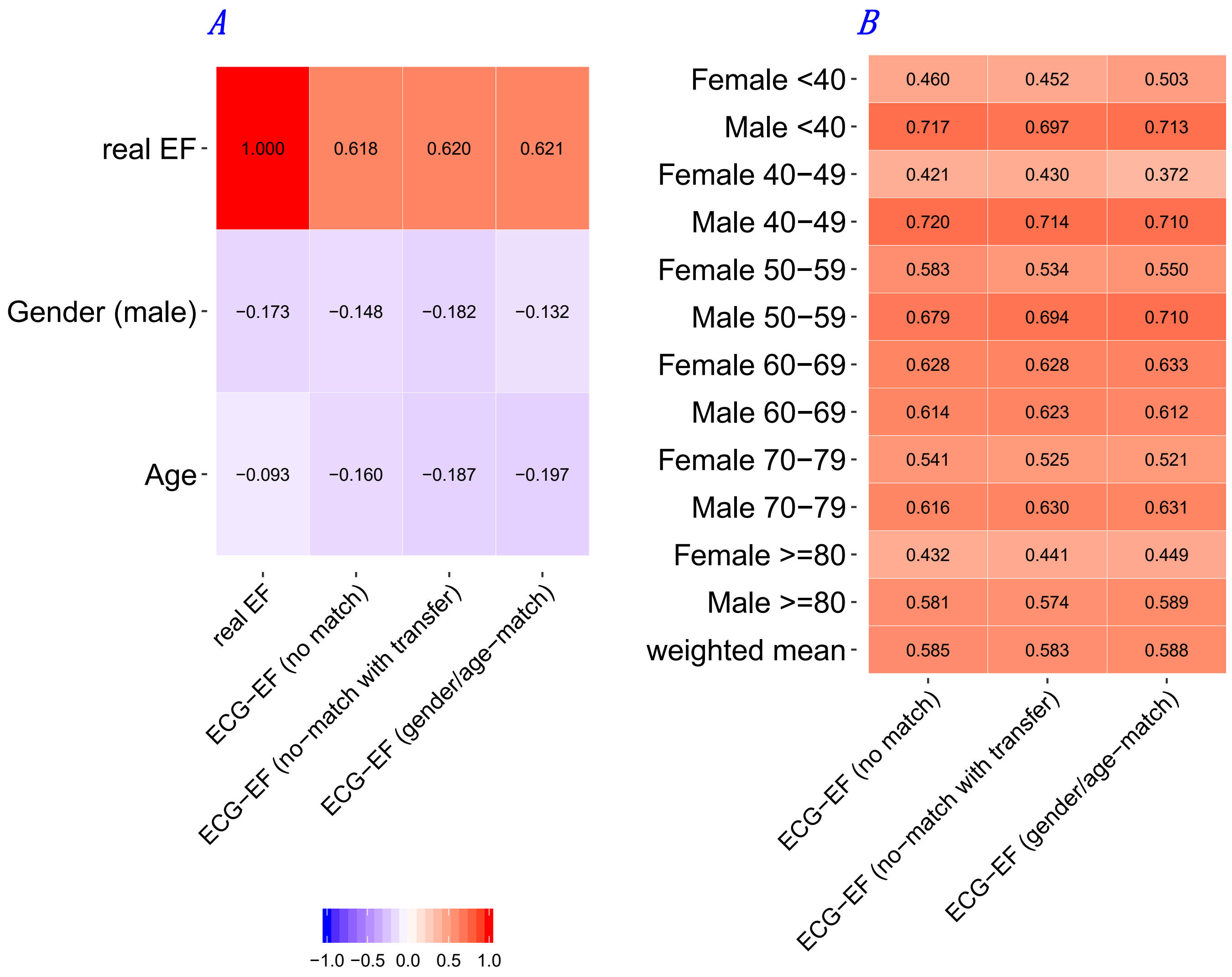

Supplementary Figure S1 | Performance comparison of DLMs trained by 3 different training strategies in validation cohort. The ECG-EF (…) were made by the predictions of DLMs using different strategies. The heatmaps were colored according to the values of correlation. The gender/age-matching strategy provides the highest crude correlation between estimated EF and real EF (left panel, A). The values in stratified analysis (right panel, B) were the correlations between estimated EF and real EF in each subgroup, which also shows the better weighted mean of correlations (weighting by number of samples) in gender/age-matching strategy.

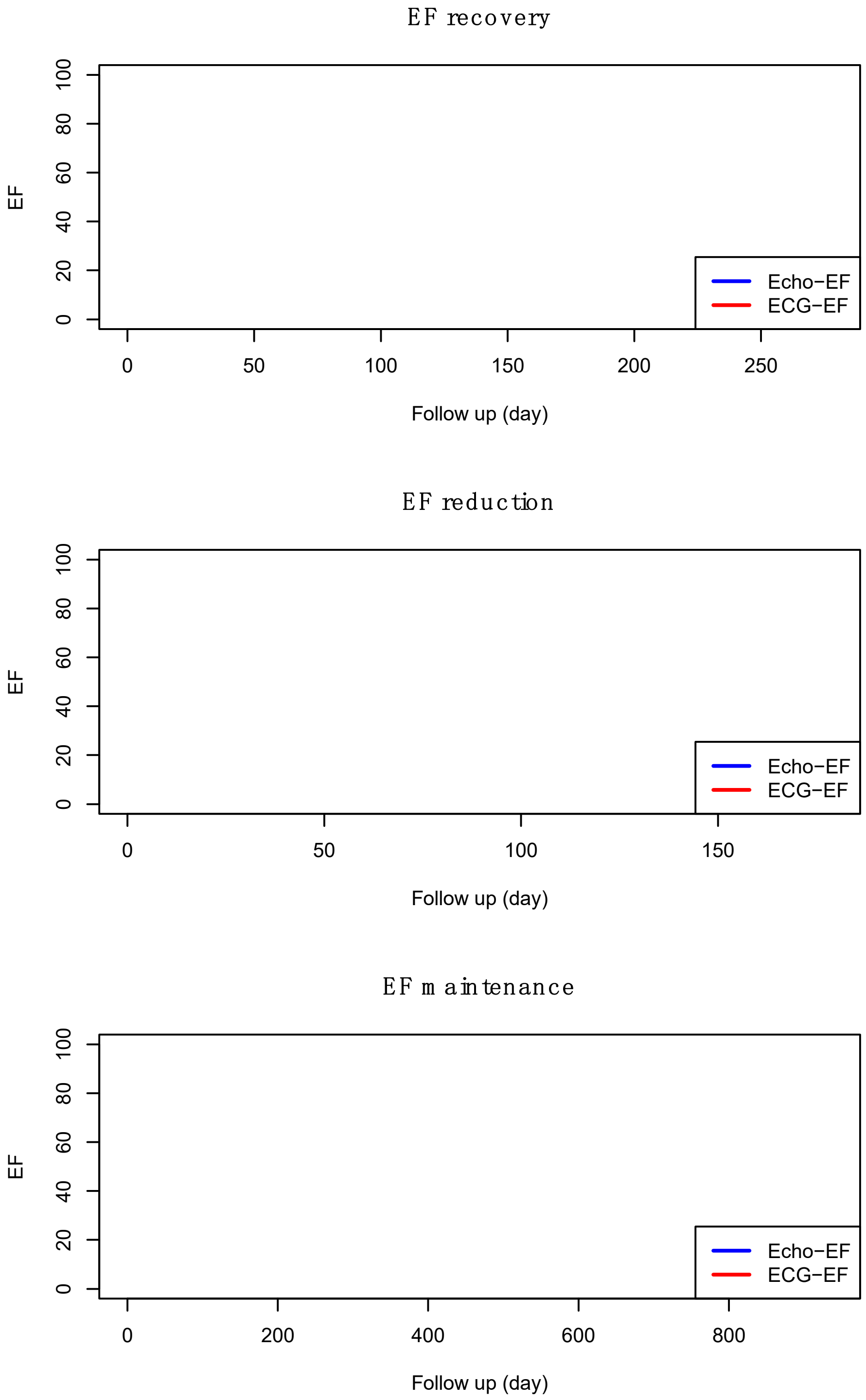

Supplementary Figure S2 | The selected cases with more than 3 ECHO-EFs and ECG-EFs. The trend lines were based on the linear regression

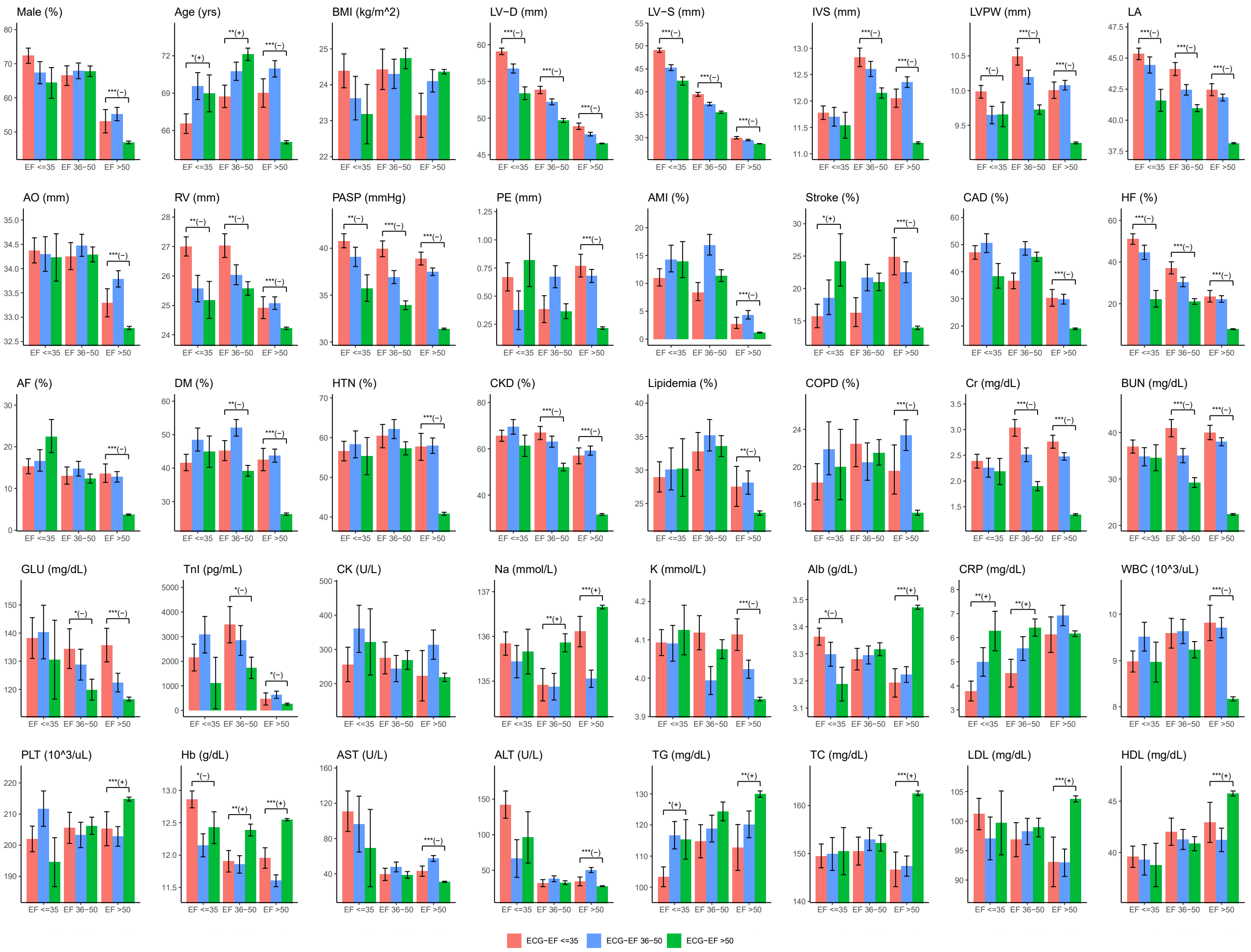

Supplementary Figure S3 | The patient characteristics in different ECG-EF groups and real EF groups. The bars represent the mean or proportion where appropriate and corresponding 95% conference intervals, which are adjusted by real EF in each EF group via linear or logistic regression. The significant tests are based on the trend test (*: p for trend < 0.05; **: p for trend < 0.01; ***: p for trend < 0.001), and the sign represents the correlation direction.

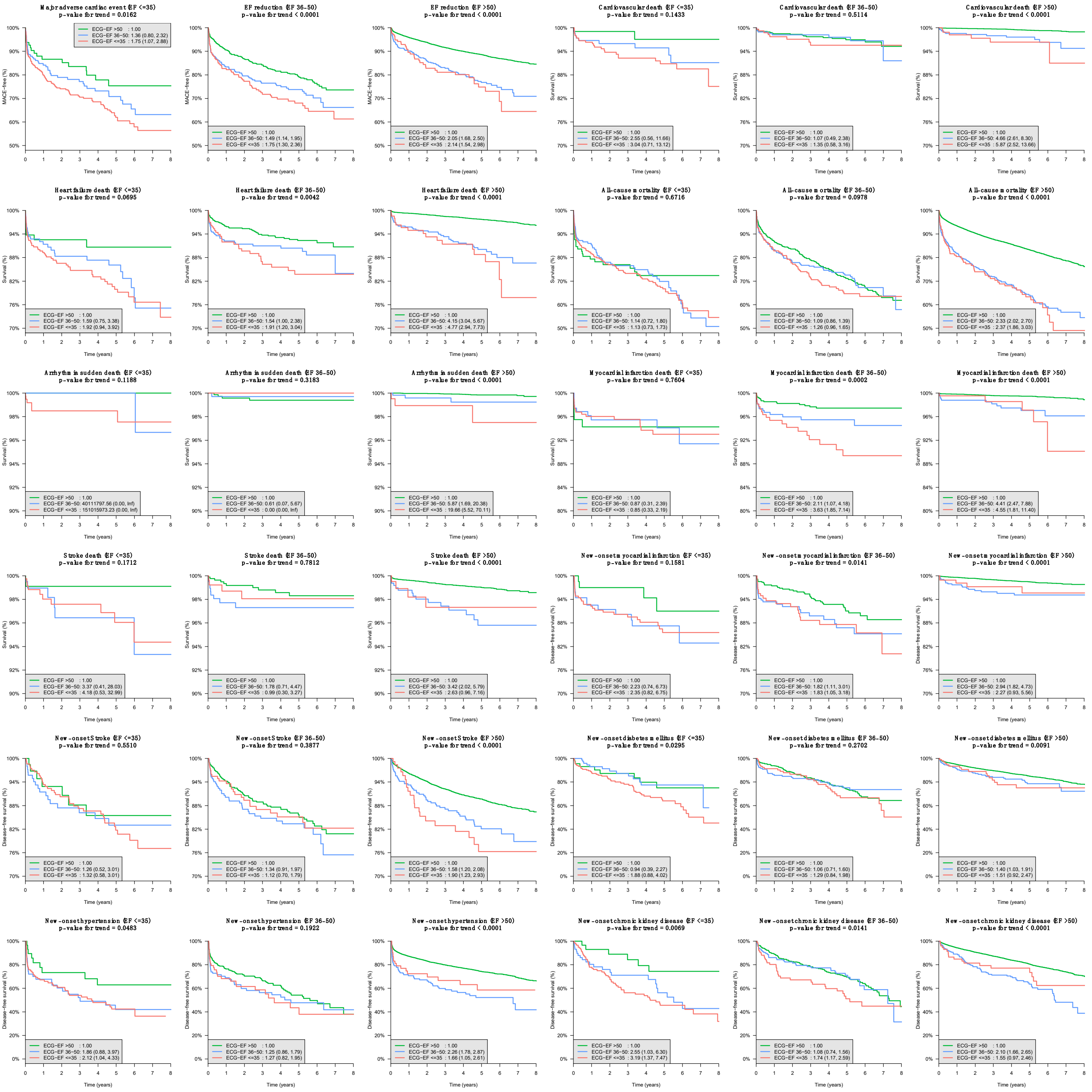

Supplementary Figure S4 | The comparison between each ECG-EF group on secondary outcomes. All analyses were based on Cox proportional hazard model adjusting the ECHO-EF.

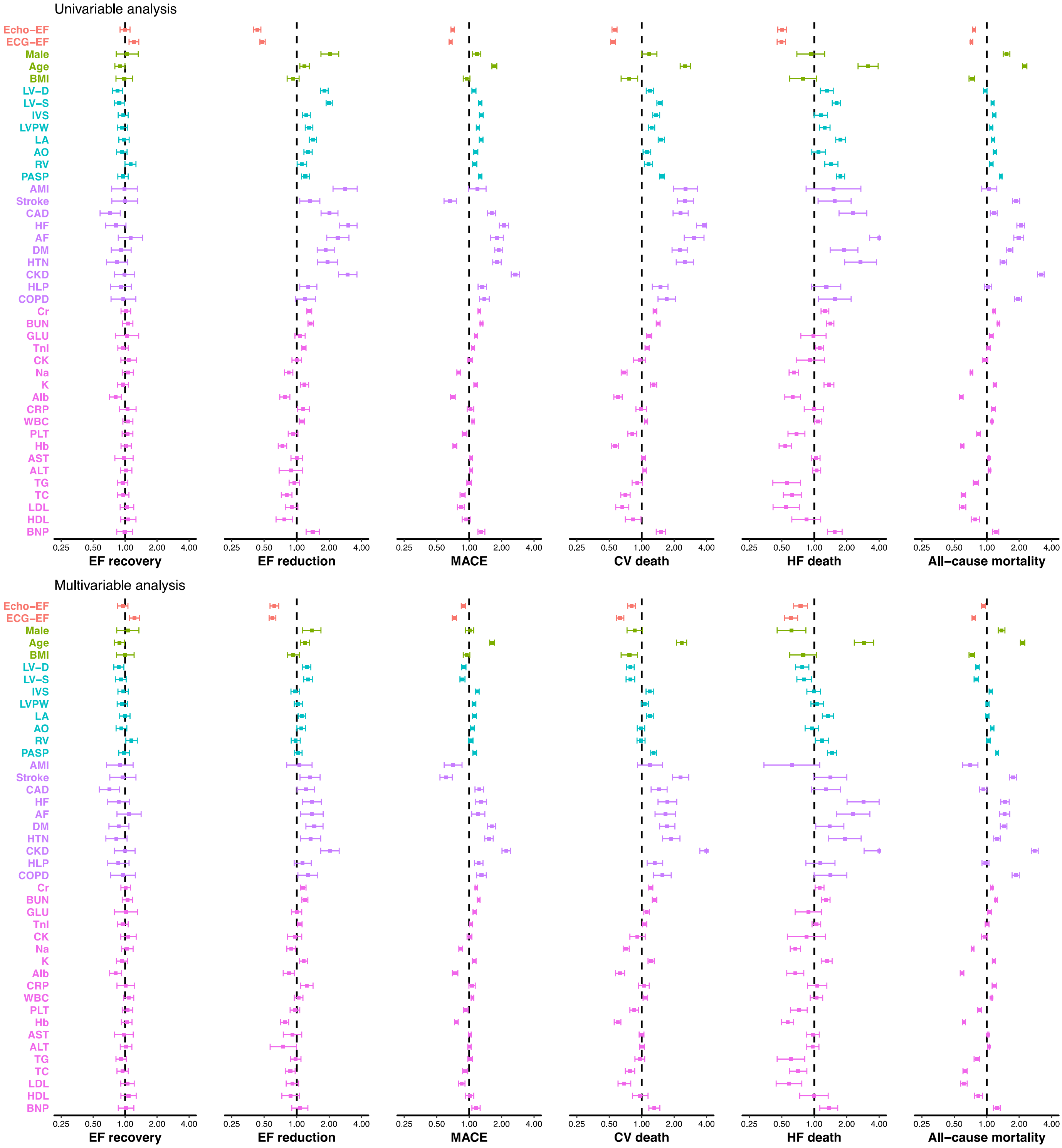

Supplement Figure S5 | Risk effect analysis of patient characteristics on primary outcomes. The univariable and multivariable analysis are conducted by Cox proportional hazard model, and the adjusted variables include ECHO-EF and ECG-EF. The continuous variables are standardized by mean and standard deviation, so the units of each continuous variable were 1 standard deviation.

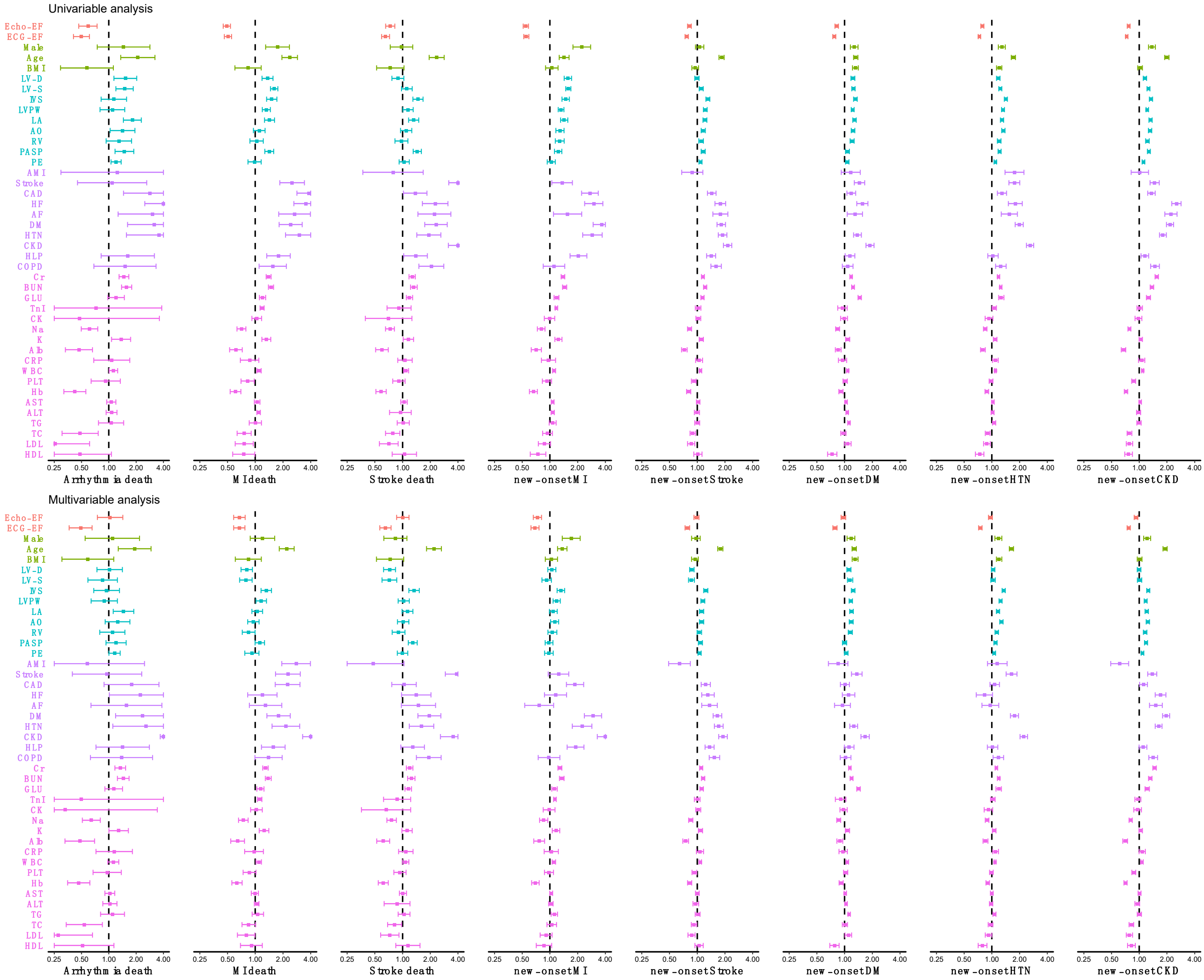

Supplementary Figure S6 | Risk effect analysis of patient characteristics on secondary outcomes. The univariable and multivariable analysis are conducted by Cox proportional hazard model, and the adjusted variables include ECHO-EF and ECG-EF. The continuous variables are standardized by mean and standard deviation, so the units of each continuous variable were 1 standard deviation.

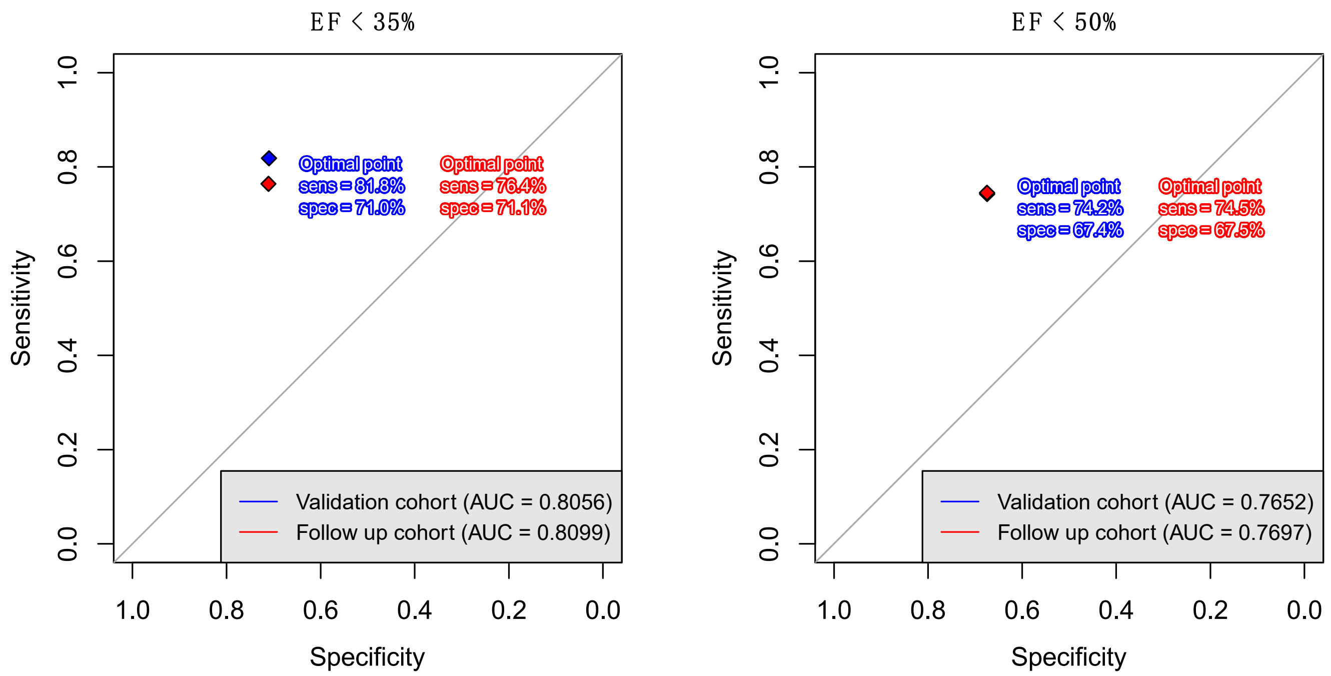

Supplement Figure S7 | The performance for detection of decreased EF using BNP in validation and follow-up cohorts. ROC curves demonstrate two cut-off points (EF≤35% and EF≤50%) to calculate the sensitivities and specificities. The optimal point was based on the maximum of Yuden index in validation cohort.

Supplementary Table S1 | Corresponding patient characteristics and laboratory results of AD and non-AD records in the ECG dataset

	Development cohort			Validation cohort			Follow-up cohort			p value†
	EF > 35 (*n* = 53,428)	EF ≤ 35 (*n* = 3778)	p value	EF > 35 (*n* = 10,206)	EF ≤ 35 (*n* = 556)	p value	EF > 35 (*n* = 19,889)	EF ≤ 35 (*n* = 740)	p value	
**Demographic data**										
OPD/HC	7719(14.4%)	143(3.8%)	<0.001	1617(15.8%)	22(4.0%)	<0.001	3191(16.0%)	60(8.1%)	<0.001	<0.001
Sex (male)	28908(54.1%)	2892(76.5%)	<0.001	5448(53.4%)	398(71.6%)	<0.001	9743(49.0%)	516(69.7%)	<0.001	<0.001
Age (years)	66.6 ± 16.8	67.2 ± 14.9	0.015	65.9 ± 17.6	66.6 ± 16.5	0.329	65.8 ± 16.8	67.8 ± 15.9	0.001	<0.001
BMI (kg/m^2)	24.8 ± 6.1	24.6 ± 7.1	0.101	24.7 ± 7.6	24.9 ± 4.9	0.700	25.0 ± 34.8	24.3 ± 5.1	0.798	0.502
Smoking	371(35.5%)	32(65.3%)	<0.001	67(37.9%)	0(0.0%)	0.529	109(37.6%)	1(33.3%)	1.000	0.966
SBP (mm Hg)	140.7±31.1	134.6±29.3	<0.001	134.4±26.8	151.7±5.8	0.178	133.3±27.6	115.7±21.4	0.213	0.168
DBP (mm Hg)	78.7±20.2	83.6±21.6	<0.001	77.5±17.3	102.3±9.8	0.023	80.4±18.7	80.7±21.4	0.807	0.908
**Echocardiography data**										
EF (%)	64.3 ± 10.1	26.4 ± 6.3	<0.001	65.4 ± 10.0	25.7 ± 6.5	<0.001	66.0 ± 9.1	26.1 ± 6.2	<0.001	<0.001
LV-D (mm)	47.2±6.9	57.0±8.8	<0.001	46.8±6.9	58.3±9.5	<0.001	47.0±6.6	57.6±9.2	<0.001	<0.001
LV-S (mm)	29.8±5.8	46.3±9.9	<0.001	29.4±5.8	47.9±10.4	<0.001	29.3±5.5	47.0±10.2	<0.001	<0.001
IVS (mm)	11.4±2.7	11.3±2.6	0.053	11.3±2.5	11.4±2.6	0.741	11.3±2.6	11.7±2.6	<0.001	0.015
LVPW (mm)	9.4±1.7	9.7±1.7	<0.001	9.3±1.7	9.9±2.2	<0.001	9.3±1.8	9.8±1.8	<0.001	0.062
LA (mm)	38.7±7.5	42.6±8.3	<0.001	38.6±7.6	44.8±9.0	<0.001	38.6±7.3	44.5±9.4	<0.001	0.138
AO (mm)	33.0±4.3	34.0±4.6	<0.001	32.9±4.3	33.6±4.5	<0.001	32.9±4.3	34.3±5.1	<0.001	0.001
RV (mm)	23.5±4.8	24.6±5.7	<0.001	24.6±5.2	26.2±5.9	<0.001	24.3±5.3	26.4±6.1	<0.001	<0.001
PASP (mm Hg)	33.9±11.5	38.9±14.6	<0.001	33.0±11.2	40.5±15.6	<0.001	32.0±10.5	39.5±15.1	<0.001	<0.001
**Disease history**										
AMI	5283(9.9%)	1052(27.8%)	<0.001	735(7.2%)	108(19.4%)	<0.001	722(3.6%)	128(17.3%)	<0.001	<0.001
Stroke	9705(18.2%)	691(18.3%)	0.847	1996(19.6%)	109(19.6%)	0.978	3253(16.4%)	148(20.0%)	0.009	<0.001
CAD	17339(32.5%)	2313(61.2%)	<0.001	3198(31.3%)	321(57.7%)	<0.001	5100(25.6%)	445(60.1%)	<0.001	<0.001
HF	6203(11.6%)	2005(53.1%)	<0.001	1269(12.4%)	328(59.0%)	<0.001	2450(12.3%)	453(61.2%)	<0.001	0.184
AF	5165(9.7%)	687(18.2%)	<0.001	934(9.2%)	101(18.2%)	<0.001	1288(6.5%)	161(21.8%)	<0.001	<0.001
DM	19671(36.8%)	1821(48.2%)	<0.001	3441(33.7%)	235(42.3%)	<0.001	6063(30.5%)	349(47.2%)	<0.001	<0.001
HTN	25657(48.0%)	2078(55.0%)	<0.001	5040(49.4%)	332(59.7%)	<0.001	9795(49.2%)	494(66.8%)	<0.001	<0.001
CKD	24139(45.2%)	2779(73.6%)	<0.001	4243(41.6%)	391(70.3%)	<0.001	7126(35.8%)	506(68.4%)	<0.001	<0.001
HLP	16966(31.8%)	1268(33.6%)	0.021	2866(28.1%)	158(28.4%)	0.864	4899(24.6%)	218(29.5%)	0.003	<0.001
COPD	7824(14.6%)	615(16.3%)	0.006	1748(17.1%)	103(18.5%)	0.395	3180(16.0%)	145(19.6%)	0.009	<0.001
**Laboratory test**										
eGFR (mL/min/1.73m^2)	73.1±40.1	55.0±37.0	<0.001	73.0±39.2	56.8±32.9	<0.001	76.8±37.4	54.5±32.0	<0.001	<0.001
Cr (mg/dL)	1.6±2.1	2.3±2.3	<0.001	1.6±2.2	2.0±1.9	<0.001	1.5±2.0	2.3±2.7	<0.001	<0.001
BUN (mg/dL)	28.5±25.4	36.9±26.8	<0.001	29.0±27.2	36.5±26.2	<0.001	24.1±22.0	36.0±26.8	<0.001	<0.001

	Development cohort			Validation cohort			Follow-up cohort			p value†
	EF > 35 (*n* = 53,428)	EF ≤ 35 (*n* = 3778)	p value	EF > 35 (*n* = 10,206)	EF ≤ 35 (*n* = 556)	p value	EF > 35 (*n* = 19,889)	EF ≤ 35 (*n* = 740)	p value	
GLU (gm/dL)	120.4±50.7	126.6±52.9	<0.001	116.5±45.6	116.6±41.4	0.978	117.7±52.6	137.6±80.9	<0.001	<0.001
TnI (pg/mL)	1723.1±8027.8	5692.2±16590.8	<0.001	1113.9±6900.0	3033.9±12212.5	<0.001	640.3±5201.6	2271.9±9613.8	<0.001	<0.001
CK (U/L)	328.6±1189.2	559.8±1619.1	<0.001	305.4±1122.2	276.9±960.4	0.575	232.6±894.8	296.2±890.2	0.098	<0.001
Na^+ (mmol/L)	137.2±5.3	137.5±5.6	0.002	136.5±5.5	136.2±5.2	0.344	136.5±5.0	135.7±5.2	<0.001	<0.001
K^+ (mmol/L)	4.0±0.6	4.1±0.7	<0.001	4.0±0.6	4.1±0.8	<0.001	4.0±0.6	4.1±0.7	<0.001	0.624
Alb (g/dL)	3.4±0.6	3.3±0.5	<0.001	3.3±0.6	3.4±0.5	0.050	3.4±0.6	3.3±0.6	<0.001	<0.001
CRP (mg/L)	5.6±7.4	5.1±6.6	0.001	6.4±7.8	4.8±6.5	<0.001	6.2±7.6	4.5±6.1	<0.001	<0.001
WBC (10^3/μL)	9.4±7.3	10.4±6.2	<0.001	9.5±7.3	9.9±6.4	0.270	8.4±5.5	9.1±4.4	<0.001	<0.001
PLT (10^3/μL)	225.9±92.0	209.5±90.9	<0.001	213.1±85.8	205.4±78.2	0.039	213.4±79.2	203.7±80.7	0.001	<0.001
Hb (g/dL)	12.3±2.5	12.2±2.7	0.013	12.5±2.5	13.0±2.8	<0.001	12.5±2.3	12.6±2.6	0.203	<0.001
AST (U/L)	45.6±158.7	136.6±374.5	<0.001	48.4±190.7	70.4±181.0	0.008	32.8±87.1	100.4±451.6	<0.001	<0.001
ALT (U/L)	36.2±126.3	108.6±352.5	<0.001	41.0±182.0	111.1±379.6	<0.001	29.0±85.8	112.6±366.1	<0.001	<0.001
TG (g/dl)	130.4±105.0	114.8±71.5	<0.001	126.2±107.5	98.7±54.0	<0.001	128.4±97.2	109.0±60.5	<0.001	<0.001
TC (g/dl)	156.5±48.5	140.9±47.8	<0.001	155.3±47.9	147.6±42.2	<0.001	160.4±47.9	149.8±46.2	<0.001	<0.001
LDL (g/dl)	92.4±38.1	85.2±37.5	<0.001	98.4±36.6	96.5±36.1	0.336	102.5±38.1	99.8±39.6	0.167	<0.001
HDL (g/dl)	41.8±15.0	36.8±13.3	<0.001	43.4±13.5	36.6±12.3	<0.001	45.0±14.3	39.4±12.8	<0.001	<0.001
BNP (pg/mL)	555.6±900.8	1735.0±1488.4	<0.001	579.4±911.1	1742.0±1555.0	<0.001	564.1±872.1	1655.1±1409.9	<0.001	0.958

†: hypothesis test of the differences among the development cohort, validation cohort, and follow-up cohort; Abbreviations: EF, ejection fraction; OPD/HC, outpatient department/health center; BMI, body mass index; SBP, systolic blood pressure; DBP, diastolic blood pressure; LV-D, left ventricle (end-diastole); LV-S, left ventricle (end-systole); IVS, Interventricular septum; LVPW, left ventricular posterior wall; LA, left atrium; AO, aortic root; RV, right ventricle; PASP, pulmonary artery systolic pressure; PE, pericardial effusion; AMI, acute myocardial infarction; CAD, coronary artery disease; HF, heart failure; AF, atrial fibrillation; DM, diabetes mellitus; HTN, hypertension; CKD, chronic kidney disease; HLP, hyperlipidemia; COPD, chronic obstructive pulmonary disease; Cr, creatinine; BUN, blood urea nitrogen; GLU, fasting glucose; TnI, troponin I; CK, creatine kinase; Na^+, sodium; K^+, potassium; Alb, albumin; CRP, C-reactive protein; WBC, white blood cell count; Hb: hemoglobin; AST, aspartate aminotransferase; ALT, alanine aminotransferase; TG, triglyceride; TC, total cholesterol; LDL, low density lipoprotein cholesterol; HDL, high density lipoprotein cholesterol; BNP, brain natriuretic peptide.
